# Countermeasures for Maintaining Cardiovascular Health in Space Missions

**DOI:** 10.2174/1573403X19666230330083225

**Published:** 2023-07-17

**Authors:** Jhilam Pramanik, Akash Kumar, Lakshay Panchal, Bhupendra Prajapati

**Affiliations:** 1 Department of Food Technology, ITM University, Gwalior, Madhya Pradesh, India;; 2 Department of Food Technology, SRM University, Sonipat, Haryana, India;; 3 Maharishi Markandeshwar Institute of Physiotherapy and Rehabilitation, Maharishi Markandeshwar University, Mullana, Haryana, India;; 4 Shree S.K. Patel College of Pharmaceutical Education and Research, Ganpat University, India

**Keywords:** Space mission, cardiac health, exercise, medicinal, artificial gravity, nutritional

## Abstract

During space exploration, the human body is subjected to altered atmospheric environments and gravity, exposure to radiation, sleep disturbance, and mental pressures; all these factors are responsible for cardiovascular diseases. Under microgravity, the physiological changes related to cardiovascular diseases are the cephalic fluid shift, dramatic reduction in central venous pressure, changes in blood rheology and endothelial function, cerebrovascular abnormalities, headaches, optic disc edema, intracranial hypertension, congestion of the jugular vein, facial swelling, and loss of taste. Generally, five countermeasures are used to maintain cardiovascular health (during and after space missions), including shielding, nutritional, medicinal, exercise, and artificial gravity. This article concludes with how to reduce space missions' impact on cardiovascular health with the help of various countermeasures.

## 1 INTRODUCTION

In space missions, the human body is exposed to various physical and psychological stresses, such as prolonged exposure to microgravity, decreased physical activity, lack of sleep, nutrition restrictions, circadian changes, ionizing radiation, and confinement and isolation [1]. Space has extreme climatic conditions that may harm cardiovascular health [2,3]. As the scope of commercial spaceflight rises, more people will get exposed to the space environment. Altered atmospheric conditions and gravity, exposure to radiation, sleep disturbance, and mental pressures are all factors that affect cardiovascular health during space flight [4, 5]. Astronauts must use different countermeasures (nutritional, medicinal, exercise, artificial gravity) to maintain cardiovascular health during and after space missions [6]. This review will focus on the impact of various factors that can cause cardiovascular diseases and the countermeasure to maintain cardiovascular health during and after a space mission.

## 2 IMPACT OF MICROGRAVITY ON CARDIOVASCULAR HEALTH

Cardiovascular changes start when an astronaut is released from Earth's gravitational force. In space, 1.9 L of fluid shifts from the lower body to the upper body [7]. The cephalic fluid shift causes jugular vein congestion, facial swelling, and loss of taste [8]. The fluid shift gets reversed quickly once the person reaches the earth. The intravascular fluid shift is restored within 90 minutes after reaching the earth [7, 9]. Astronauts also suffered from “chicken legs” due to cephalad fluid shifts brought on by microgravity, which are caused by a drop in the movement and intrathoracic pressure of about two liters of fluid from the legs [10, 11].

The arterial blood pressure changes above and below the heart while standing up straight under the ordinary gravity of Earth. However, uniform arterial pressure is experienced under the microgravity environment, which lessens the physiological necessity for the body's blood pressure control systems and, as a result, lessens the stress on the heart [2, 12]. During the initial twenty hours of spaceflight, astronauts may undergo increased heart chamber volumes, increased atrial diameter, and a drop in central venous pressure [10, 13]. Parabolic flights showed that central venous pressure decreased in 8 astronauts by 1.3 mm Hg during weightlessness, while the left atrial diameter increased by 3.6 mm. There is a fall in oesophageal pressure by 5.6 mm Hg. This could help to explain why atrial diameter increased even though central venous pressure decreased [14, 15].

The cardiovascular system must adjust to the enormous fluid shift brought on by the absence of the blood pressure gradient in the lower and upper body. The body's physiological response to the enormous fluid translocation is crucial. The stimulation of baroreceptors by the distension of the heart and the presence of fluid that has been redistributed inhibits the renin-angiotensin-aldosterone pathway and increases atrial natriuretic peptide production. This is the most significant effect [2, 16]. The sum of these reactions causes a ten to fifteen percent drop in blood plasma volume. It is significant to highlight that these reductions in plasma volume are likely caused by elevated vascular pressures in the upper body and decreased interstitial pressures, which combined promote transcapillary fluid migration into the interstitium of the upper body [16, 17]. Notably, prolonged spaceflight has also caused cardiac remodeling [18]. Research indicated that after only 10 days in space, the left ventricular mass of four male astronauts decreased by an average of 12% ± 6.9% [19, 20]. Individuals lost ten to fifteen percent of their hematocrit as measured shortly after landing at the end of short-duration space journeys lasting ten to fourteen days, which is equivalent to a loss of 1% RBC mass each day [21, 22].

According to Feger *et al.*, *in vitro* research on rat cardiomyocytes, microgravity leads to physiological adaptation through changes in the composition of protein and the function of endoplasmic reticulum (ER), ribosomes, and mitochondria, which is responsible for less synthesis of protein and leads to atrophy. It seems that cardiomyocytes exposed to microgravity experience a decrease in protein translation through the rough endoplasmic reticulum (ER) [23]. This research also revealed a considerable decline in tropomyosin and myosin regulatory light chain, two cytoskeletal proteins essential for mitochondrial localization and indicative of muscle atrophy [23].

Cardiac arrhythmias are another issue that worries NASA. Typical arrhythmias include premature contractions, chaotic regional depolarization, atrial or ventricular fibrillation, bradycardia, tachycardia, and slower-than-normal or faster-than-normal heart rate [24]. As a result of cardiac arrhythmias, the risk of sudden cardiac death, stroke, cardiovascular illness, and dementia is increased [25]. Astronaut James Benson Irwin first experienced premature ventricular contractions associated with hypokalemia during the Apollo 15 program (1961-1972). Subsequently, multiple occurrences of cardiac arrhythmias were documented during the Skylab programs (1973-1979) [19].

Orthostatic intolerance might be fatal upon re-entering into a gravitational field in the case of an emergency evacuation. On Earth, several factors contribute to orthostatic intolerance, including decreased plasma and stroke volumes and constricted restriction arteries [10]. Astronauts subjected to long-duration spaceflight (129-190 days) than in controls who have only experienced short-duration spaceflight. According to some investigations, 83% of astronauts returning from long-duration flights and 20% to 30% of astronauts returning from short-duration flights exhibit symptoms of orthostatic intolerance [26, 27]. Despite NASA giving astronauts a fluid-loading regimen before re-entry, it has been shown that the amount of terrestrial plasma is nevertheless decreased by 7% to 20% compared to pre-flight [27]. During a space mission, the mean internal jugular vein area of 11 astronauts was assessed by ultrasonography. The area of the jugular vein was found to be increased sevenfold as a result of cerebrospinal fluid alterations [28]. In weightlessness, a change in blood rheology and endothelial functional increase the risk of IJ vein thrombosis [28, 29]. Lower body negative pressure (LBNP) of 20-40 mmHg has been shown in ground-based models to reduce cephalad fluid excess and intracranial hypertension [30]. LBNP prevented internal jugular and portal vein dilatation during long-duration spaceflight [31].

## 3 IMPACT OF RADIATION ON CARDIOVASCULAR HEALTH

Weather and cosmic radiation risk to the heart are crucial for long-term spaceflight. Cardiovascular disease (CVD) and stroke are thought to have contributed to one-third of the ionizing radiation (IR)-induced mortality after the atomic bombs of Japan [32]. Additionally, it is well-recognized that ionizing radiation (RT) causes CVD in cancer patients [33]. It is generally known that large doses of ionizing radiation may cause cardiac disease in healthy people as well. This illness manifests as arrhythmia, valve abnormalities, conduction defects, myocardial fibrosis, and accelerated atherosclerosis, with cellular damage commencing [34, 35]. A person flying to Mars is expected to get a cumulative radiation exposure of 0.5 and 1.0 Sieverts (Sv) [36]. Thus, Mars exploration of 1000 days (600 days on Mars' surface and 400 days in space) would raise a 40-year-old man's lifetime probability of dying from radiation exposure by 1.3% to 13% [19]. Additionally, the kind of radiation present in space-protons and, beyond the Van Allen belts, HZE particles-differs from that often encountered on Earth and is hypothesized to have more physiologically harmful effects than X-rays or gamma-rays [2]. On the way to Mars, a high-energy proton will pass through the nucleus of every cell in the body; therefore, it is very important to research how proton radiation affects the heart [37].

### 3.1 Impact of Sleep Disturbance on Cardiovascular Health

Sleep and circadian rhythm changes might potentially alter blood pressure rhythmicity. Research on MIR cosmonauts found an increase in systolic blood pressure during sleep but no change in average systolic blood pressure across 24 hours [38]. Similar discoveries have been made during spaceflight [39]. On the other hand, nightly blood pressure lowering was well-preserved in astronauts on long-term ISS deployments [40].

### 3.2 Impact of Isolation and Confinement on Cardiovascular Health

Isolation and confinement are examples of psychological stressors. They induce various psychological and physical effects, such as motivational decline, fatigue, somatic complaints, and social tensions [41]. Ning *et al.* demonstrated in a population-based study that social isolation (both objective and the perception of social isolation) is correlated with a higher risk of mortality and a clear risk factor for developing CVD [42]. The effects of loneliness and social stress are chronic and develop over time. The proposed underlying mechanisms are chronic overactivation of the sympathetic nervous system and physical inactivity. There are numerous sources of stress during spaceflight, such as isolation, confinement, and separation from Earth. However, noise and vibrations associated with normal vehicle system operations or fear of equipment failure are also consistent sources of stress [43]. These stressors continue throughout the mission and may be exacerbated by interpersonal stressors and homesickness [44]. The Mars500 psychosocial experiment simulated a manned spaceflight mission to Mars that consisted of a crew of six people that spent 520 days in isolation and confinement [45]. The Mars500 study showed significant disruptions in circadian heart rate (HR) and heart rate variability (HRV). A sympathetic predominance characterizes the circadian rhythm during the waking periods, and a parasympathetic predominance during the night. The Mars105 study showed a reduced mean HR during the daytime compared with the night-time measurements during sleep, emphasizing increased parasympathetic activity during the waking periods [46].

### 3.3 Impact of Hypergravity on Cardiovascular Health after Space Mission

Astronauts who return to Earth frequently have orthostatic intolerance, especially during longer-duration missions. While orthostatic intolerance and orthostatic syncope are manageable on Earth, they might have catastrophic implications upon arriving on another celestial body [27]. Over the years, several countermeasures that can be implemented before landing have been studied. These countermeasures include salt loading, increased fluid intake, compression clothing, and medicines such as the mineralocorticoid fludrocortisone [47, 48]. Supine means arterial pressure dropped from 93 mmHg at 1g to 88 mmHg at 0g during parabolic flights [49]. This discovery was validated in subsequent parabolic flying studies [50, 51]. Because of the fast shift from weightlessness to hypergravity, data taken during parabolic flights must be interpreted carefully.

### 3.4 Cardiovascular Disease Clinical Prediction Models

Several risk assessment tools have been developed to guarantee the individual's health and quality of life. Clinical prediction models (CMP) have been subjected to several evaluations [52, 53]. Most clinical prediction models for cardiovascular disease aim to estimate the potential patients who would benefit most from early primary preventive therapy, and as a result, these models often provide a 10-year risk score [54]. NASA has unique requirements for using CPMs. Those requirements were considered, and several models were presented in Table **1**.

## 4 COUNTERMEASURES TO MAINTAIN THE CARDIOVASCULAR HEALTH

### 4.1 Medicinal

In clinical practices, different compounds such as beta-adrenergic blockers are used to prevent the activation of cardiac mechanoreceptors, fludrocortisone or electrolytes are used to increase the volume of blood in circulation, disopyramide to prevent vasovagal syncope, alpha-adrenergic agonists are used to increasing the venous return and venous tone [65]. Hypovolemia caused by microgravity leads to orthostatic impairment after space flight. To counteract this impact, US astronauts take a max of 8 pills (containing 1-g of salt) with around 912 milliliters of liquid designed to make an isotonic saline beverage 2 hours before re-entry to restore the volume of blood [66]. Angiotensin-converting enzyme inhibitors may lessen the cardiopulmonary system's harm from radiation. Rats with the heart exposed to proton radiation have tachypnea relief after receiving captopril [67]. After 32 weeks of radiation exposure, rats were given captopril, and the experimental antioxidant EUK-207 exhibited almost no molecular radiation effects [68]. Following receiving a brief course of enalapril for up to 2 weeks (10 Gy) or one month (13 Gy) after the first radiation, survival in rats exposed to 10 or 13 Gy of thoracic radiation improved. Enalapril-treated rats also decreased cholesterol-containing clefts in the alveoli, a histopathological indicator of radiation injury [69]. In space, Internal jugular (*IJ*) *vein thrombosis was* treated with heparin [70].

Vernikos and his colleagues (1991) documented that the administration of fludrocortisone helps restore plasma volume [71]. Beta-adrenergic receptor antagonists have also shown potential as radiation defenders, modulators, and mitigators, although the mechanism is uncertain. Although there have not been many preclinical investigations on beta blockers with radiation up to this point, clinical record evaluations suggest that inadvertent beta blocker usage could improve prognosis. Patients undergoing radiation treatment for non-small-cell lung cancer while taking beta blockers had substantial gains in overall survival, disease-free survival, and distant metastasis-free survival [72, 73]. Beta-blockers often have negative side effects (fatigue and dizziness). This is a concern because of the neurosensory adjustments astronauts must make while changing between various gravity states. Beta-blockers may increase respiratory distress in people with asthma and disguise the symptoms of hypoglycemia in diabetics (a risk smaller for the group of physically fit astronauts than for the growing population of space tourists) [74]. Before considering beta blockers in astronauts, most likely as a preventative medication, much more research is required.

Calcium channel blockers are efficient mitigators of vascular alterations brought on by oxidative stress in disease processes like atherosclerosis and act as radiation protection agents. They block low-density lipoprotein oxidation at high doses and lower quantities; they have a direct protective impact on cells (mechanism unclear) [75]. Although calcium channel blockers have shown effectiveness in other tissues, they have not yet been investigated as radioprotectants of cardiovascular tissues. Radiation exposure may cause taste aversion and appetite suppression. With absorbed dosages of 1 Gy, rats demonstrated a substantial taste aversion to a saccharin solution. Diltiazem, a calcium channel blocker, similarly produced dose-dependent taste aversion at dosages equivalent to or more than 10 mg/kg. Rats exposed to radiation and modest doses of diltiazem (5 mg/kg) exhibit less taste aversion, nevertheless [76]. The calcium channel blockers diltiazem, nifedipine, and nimodipine are delivered in various ways to protect against radiation-induced death. Additionally, when calcium channel blockers are coupled with zinc aspartate or dimethyl sulfoxide, the protective benefits, as shown by overall survival, seem to be synergistic [77].

Vasodilation is the cause of most calcium channel blocker adverse effects [78]. During the two initial weeks under microgravity, astronauts are prone to edema. Although calcium channel blockers could help lower taste aversion and appetite suppression linked to acute exposure in the astronaut population, this small advantage is probably too minor to give additional research a high priority. Calcium channel blockers must demonstrate effectiveness in other organ systems or more mortality trials before being seriously considered as an acute therapeutic. There is presently no proof in favor of prophylactic usage.

### 4.2 Shielding

Radiation-induced oxidation has been found to affect human organisms [79]. Oxidative stress causes molecular changes that can lead to mutagenesis, neurocognitive impairment, higher risk of epithelial malignancies and cardiovascular disease, lower resistance to diseases, and several other health impacts known as 'premature aging [80]. The crew sleeping areas on the ISS are also coated with polyethylene, a hydrogen-rich substance that provides a 20% decrease in radiation exposure [81]. Water is another hydrogen-rich substance that can be used for shielding applications. On the ISS, a protective layer of sanitary wipes and moist cloths with an average water thickness of 6.3 g/cm2 was shown to minimize the equivalent dosage by 37% [82]. In addition to typical shielding requirements, plans for spacecraft or habitats beyond LEO include the construction of a “storm shelter”-a fully insulated place where personnel may seek refuge if a solar particle event (SPE) occurs. Based on previous research findings SPEs, an appropriate degree of protection is expected to be achievable with an aluminum equivalent of 28 g/cm2. While these shelters can reduce the dangers of SPEs, their capacity to reduce the risks of GCRs further is limited, with even ideal shielding materials only providing a 35% dosage reduction [83]. Ongoing research includes utilizing innovative materials such as hydrogenated boron nitride nanotubes with potential radiation-protective characteristics and good strength, heat resistance, and flexibility [84, 85]. Finally, attempts are being made to construct an “active shielding” option, which would include the development of a protective plasma, electrostatic, or magnetic field surrounding the space vehicle that would successfully deflect incident radiation [86].

#### 4.2.1 Nutritional

Cardiovascular problems are a major problem for astronauts, although nutrition's function in cardiovascular adaptation is still poorly understood [87, 88]. On Earth, omega-3 fatty acids have a definite positive influence on cardiovascular health, but these benefits have not been examined in space. But the early initiatives to boost astronauts' fish consumption and omega-3 fatty acids to benefit other systems (bone, muscle) will also have good impacts here [89]. According to Rizos *et al.* (2012), omega-3 fatty acids and fish positively impacted health [89]. The possible action of omega 3 to protect the cardiovascular system is shown in Fig. (**1**).

The excess salt intake on Earth causes high blood pressure and removes large amounts of water from the body [90]. Long-term negative energy balance results in impaired cardiovascular function, poor muscular performance, increased muscle fatigue, low immunity, reduced wound healing power, and disturbed sleep [91]. Chronic energy deficit can increase adverse physiological changes such as cardiovascular diseases, bone density, muscle loss, and immunodeficiency [91-94]. Space exercise increases the amount of energy required to maintain energy balance [95]. Compared to the nutritional reference intake on Earth, astronauts on the European mission toward the end of the past century had an insufficient intake of calories, fluids, and calcium, as well as excessive salt consumption. Inadequate amounts of these nutrients significantly impact hormone balance, cardiovascular health, and bone health [96].

According to research, high protein consumption combined with additional branch-chain amino acids can promote heart health [97]. Consumption of low-cholesterol foods maintains cholesterol levels and lowers the chance of heart disease. During the mission, if there is a fall in astronaut weight, we first have to raise that individual's calorie consumption. High protein consumption may also be beneficial to maintaining good health, as they reduce muscle atrophy [90].

Despite the lack of research on the ideal composition of dietary energy sources for spaceflight, a group of leading dietitians established nutritional recommendations to ensure appropriate consumption of nutrients for long-duration space missions of up to 360 days. These guidelines suggest protein consumption of 0.8 g per kg body mass, 350 g of carbohydrates, and 70 g of fats per day [98, 99]. As a result, the total daily energy requirements from the different macronutrients are equivalent to those on Earth, with 12-15% of total energy coming from protein, 50-55% from carbohydrates, and 30-35% from fat [92]. The Water Recovery System, which produces the majority of the crews' water supply, uses cabin urine, perspiration, and condensation to make clean water that is then distilled and purified to provide drinkable water [100]. While average intake is often lower than required, astronauts do not develop space-related dehydration despite the 2000 ml/day of water advised for consumption on the ISS [101]. Astronauts were reported to have a decreased nutritional intake before ISS trips, on average only consuming 70% of what was needed (except Skylab) [102]. This could be a factor in the persistently reported reduction of body mass [103]. For instance, a study of four astronauts aboard the space shuttle revealed a drop in food intake, which in turn caused a loss in body fat, suggesting a situation of negative energy balance and insufficient nutrition [104]. A change in food palatability brought on by a change in smell and flavor may also have contributed to difficulties ingesting a sufficient nutritional intake [8]. According to Heer *et al.*, space foods should not only supply enough calories but also provide other essential nutrients in an appropriate amount to prevent the negative impacts of the space environment [105].

Selenium appears to be one of the micronutrients that might be reduced during a space mission, potentially impacting oxidative damage defense. Selenium insufficiency is also linked to poor immune system function [80]. Furthermore, supplementation appears to enhance the immunological response. In two small investigations, healthy [106, 107] and immunocompromised persons [108] were treated with 200 mcg/day of selenium for 8 weeks and exhibited an improved immune response to foreign substances to those receiving a placebo. A substantial amount of fundamental research also suggests that selenium regulates the production of cell-signaling molecules known as cytokines, which organize the immunological response [109].

The presence of radioprotective food components during radiation exposure may reduce the first ionizing events caused by radiation. Many chemoprotective chemicals can be found in nature. It has been noted that such dietary supplements can reduce oxidative damage in people exposed to ionizing radiation [110-116]. Unfortunately, antioxidant substances require extremely high dosages to be clinically effective as single agents.

Therefore, getting these substances of natural origin is desirable in terms of palatability and the quality of their biological activity. However, as previously noted, it is not feasible to provide fresh meals and vegetables during a space mission; thus, new methods of obtaining these nutrients, particularly those with chemoprotective properties, must be discovered. Dietary supplements in compact tablets and various alternative delivery methods with a long shelf life, stability, and storage efficiency can be viable solutions [80]. In 2005, NASA entered into a Space Act Agreement with industry stakeholders to discover nutritional supplements and substances to support the health of astronauts. As a result of these collaborative efforts, several studies were carried out [117-119]. Consequently, a daily multivitamin and multimineral formulation were designed to assure and normalize the consumption of all micronutrients. An independent study has also been conducted to determine the efficacy of dietary products, supplements, and combinations in boosting human adaptability and minimizing the harmful effects of ionizing radiation [118]. Space foods are not nutrient-dense, so they cannot be used as alone countermeasures for muscle atrophy [120, 121], bone loss [120, 122, 123], and cardiovascular deconditioning during space missions [124-126].

## 5 EXERCISE

Long-duration space missions decreased aerobic exercise capacity and diffusive oxygen transport [127]. Peak oxygen consumption levels can be maintained for long periods in space if an individual does intensive training. Seven astronauts out of the fourteen have achieved their pre-flight peak oxygen consumption levels in space. Astronauts with maintained cardiac fitness trained harder than those with declining peak oxygen consumption. These findings highlighted the importance of exercise for maintaining cardiac health during space missions [128].

The space station has equipment for resistance workouts using the Advanced Resistive Exercise Device (ARED) and aerobic activities utilizing a treadmill or stationary bicycle. Advanced Resistive Exercise Device (ARED) helps in resistance exercise on the ISS. Two-piston/cylinder systems with changeable loads provide the resistive force. The weight for bar workouts may be adjusted from 0 to about 2,670 N. It can be loaded to roughly 670 N for cable workouts. Deadlifts, squats, heel raises, hip abduction and adduction, bench presses, biceps curls, triceps extension, and upright rows are among 29 free-weight workouts available to astronauts [129]. Advanced Resistive Exercise Device (ARED) will be housed in the new module but won't occupy a rack space in Node-3. It was created to enhance the ISS's present workout capabilities. Providing a more continuous force across the range of action replicates the properties of conventional resistive workouts (weighted bars or dumbbells). It provides regular upper and lower body workout exercises, including squats, deadlifts, heel raises, bicep curls, and bench presses [130]. Table **2** represents the exercises for maintaining cardiovascular health. To stay fit in microgravity, astronauts must exercise for around 2.5 hours daily, 6 days a week. During space missions, treadmills are used by astronauts to stay active. The space station has two treadmills: the Treadmill Vibration Isolation System (TVIS) and the Combined Operational Load Bearing External Resistance Treadmill (COLBERT) [131]. The ISS's Cycle Ergometer with Vibration Isolation System (CEVIS) provides astronauts with a low-impact, high-cardio workout. Crew members can use CEVIS to change the resistance and pace to optimize their particular training profile [132]. Furthermore, astronauts can wear special garments that exploit differential pressure to draw blood back into the abdominal area [133].

### 5.1 Artificial Gravity

Artificial gravity (AG) has long been explored and recommended as a solution for the physiological deconditioning of several organ systems [134-137]. A multi-system defense mechanism is provided in the form of artificial gravity. It involves simulating a gravitational environment that tests each physiological system much as on Earth. The physiological deconditioning induced during long-duration spaceflight, including bone loss, muscular atrophy, cardio-vascular deconditioning, neuro vestibular abnormalities, space anemia, and immune system inadequacy, is thought to be addressed by artificial gravity [138]. Artificial gravity may also lessen or eliminate physiological deconditioning while enhancing cleanliness, habitability, and medical procedures [139]. There are many ways to create AG in a spacecraft, including centrifugal force or linear acceleration. Two basic techniques-intermittent artificial gravity and continuous artificial gravity *via* spinning on an onboard small radius centrifuge-can be distinguished under the second category. Continuous artificial gravity-A big spacecraft's spin may provide continuous artificial gravity. Tsiolkovsky initially put out this idea in 1883. There are several benefits to exercising while centrifuging in a short-radius centrifuge. Increased acceleration tolerance may be a benefit of exercise when centrifuging. There is a chance that the centrifuge's physiological stress is too great, which might lead to some degree of orthostatic intolerance. Active activity during centrifugation stimulates the leg muscle pumps, which helps the venous return and prevents blood from pooling in the legs, protecting the astronauts from syncope or fainting [140]. Additionally, it has been shown that exercising and centrifugation together effectively prevent cardiovascular deconditioning [141, 142]. Few ground-based research has examined exercise combined with short-radius centrifugal intermittent artificial gravity as a complete countermeasure for long-duration spaceflight. A cycle ergometer is one of the top 5 workout machines used today. Exercise in a short-radius centrifuge (1.9 m) provides considerable physiological stress that might lessen orthostatic intolerance, as shown by Greenleaf *et al.* [141]. Iwase and his colleagues established that combining ergometric exercise with intermittent artificial gravity effectively prevents cardiovascular deconditioning [143, 144]. A short-arm centrifuge and aerobic exercise coupled with training for one week led to an increase in cardiovascular function, according to Yang *et al.* [145]. During 20 days of -6° Head Down Bed Rest, Katamaya *et al.* showed that the use of a short-arm centrifuge together with exercise training helps sustain respiratory and cardiovascular responses to upright activity [142]. Numerous studies have been conducted to find out the effect of AG on physiological conditioning, but there are some limitations as these studies were conducted under a different set of circumstances (exercise use/intensity, gravity level, gravity gradient, exposure period, and centrifuge design). Kaderka *et al.* utilized a set of different circumstances in their study. These variations were in the centrifuge's design, subject selection criteria, experiment methodology (such as exposure time, study length, g level, gravity gradient, *etc*.), and dependent measure selection [140]. These results underline the necessity for more thorough AG investigations that exclude some circumstances. Without further countermeasures, the lower Mars gravity (0.38g) may not be capable of maintaining good health [145]. Artificial gravity creates a gravitational condition that manages all physiological mechanisms exactly as on Earth. Artificial gravity will not eliminate all the risks related to space travel (radiation exposure), but it has been suggested as a solution for deconditioning, such as cardiovascular deconditioning, immune system deficiency, space anemia, and neuro vestibular disturbances [138, 146]. According to Diaz *et al.* (2015), the level of gravity affects the cardiovascular system’s response. The combination of artificial gravity and exercise may be effective in maintaining cardiovascular health. Furthermore, in addition to eliminating or reducing deconditioning, artificial gravity may enhance cleanliness, human habitation, and medical interventions [146].

## CONCLUSION

Research on the cardiovascular effects of space exploration has increased significantly over the last few years. For space agencies, this area of ​​research is important for assessing health risks to astronauts during and after long-distance space travel. To maintain heart health, various countermeasures are used, such as intake of salt tablets, low sodium intake, adequate potassium intake, a protein-rich diet, exercise, and artificial gravity. In this review, we focus primarily on the effects of microgravity on cardiovascular health and general countermeasures. Future studies should focus on developing appropriate countermeasures to keep our future astronauts fully safe.

## Figures and Tables

**Fig. (1):**
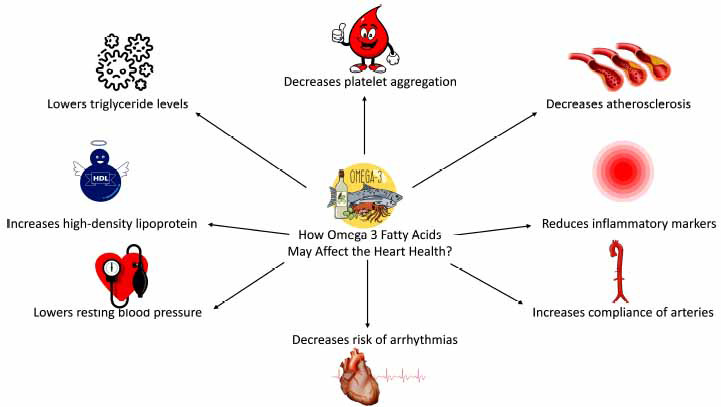
Action of omega-3 fatty acids on heart health.

**Table 1:** Clinical prediction models for the evaluation of cardiovascular disease risk.

**Clinical Risk Prediction Models**	**Outcome**	**References**
ACC/AHA ASCVD Risk Calculator	Estimates the risk of prediction of atherosclerotic cardiovascular disease	[55]
ASCVD Risk Estimator Plus	Estimates the risk prediction of atherosclerotic cardiovascular disease	[56]
Astronaut Cardiovascular Health and Risk Modification	Estimates the 10-year risk prediction of atherosclerotic cardiovascular disease	[57]
Framingham risk score	Estimates the 30-year risk prediction of cardiovascular disease	[54]
Framingham risk score	Estimates the risk of predicting cardiovascular disease and cardiovascular disease events (coronary, cerebrovascular, peripheral arterial disease, and heart failure).	[58]
LIFEtime-perspective Cardiovascular Disease	Estimates the lifetime risk and CVD-free life expectancy	[59]
QRISK	Estimates the lifetime risk prediction of cardiovascular disease	[60]
QRISK 2	Estimates the risk prediction of cardiovascular disease	[61]
QRISK 3	Estimates the risk prediction of cardiac disease and stroke.	[62]
Reynolds risk score	Estimates the risk prediction of atherosclerotic cardiovascular disease	[63]
SCORE risk estimation system	Estimation of total fatalities linked to cardiovascular risk	[64]

**Table 2:** Exercises for maintaining cardiovascular health (Data is adapted and modified from https://www.asc-csa.gc.ca/eng/astronauts/living-in-space/physical-activity-in-space.asp).

**Sr. No.**	**Space Gym’s Equipment**	**Type of Exercise**	**Method of Use**
1	ARED: Advanced Resistive Exercise Device	Targeting the muscle groups and preserving the bone density and muscle	Firmly grasp the exercise bar →. Change the resistance until it is at the proper level →. Based on the muscles you want to work on, do heel lifts, squats, and deadlifts
2	Treadmill	Cardiovascular, muscular, and skeletal	Put on the harness system → Depending on the desired level of training intensity, adjust the harness's load → Run
3	Stationary bike	Cardiovascular	Place feet on the clip pedals → To keep yourself anchored to the machine, put on the back harness → Grab the handholds to keep your balance while using the machine → Pedal

## LIST OFABBREVIATIONS

ER: Endoplasmic Reticulum
LBNP: Lower Body Negative Pressure
CVD: Cardiovascular Disease
IR: Ionizing Radiation
